# Coping during COVID-19: The Impact of Cognitive Appraisal on Problem Orientation, Coping Behaviors, Body Image, and Perceptions of Eating Behaviors and Physical Activity during the Pandemic

**DOI:** 10.3390/ijerph182111305

**Published:** 2021-10-28

**Authors:** Elisha Starick, Vanessa Montemarano, Stephanie E. Cassin

**Affiliations:** Department of Psychology, Ryerson University, Toronto, ON M5B 2K3, Canada; elisha.starick@ryerson.ca (E.S.); vanessa.montemarano@ryerson.ca (V.M.)

**Keywords:** COVID-19, body image, eating behaviors, physical activity, coping, cognitive appraisal, problem orientation

## Abstract

Large surveys indicate that many people perceive that their health behaviors (i.e., eating behaviors, physical activity, and self-care routines) and body image have changed during COVID-19; however, large individual variation exists. A person’s cognitive appraisal of COVID-19 disruptions may help account for individual differences. Those with a negative problem orientation perceive problems as “threats”, whereas those with a positive problem orientation reframe problems as “opportunities”. The present experimental study examined the impact of appraisals, specifically being prompted to reflect on the changes in health routines precipitated by COVID-19 restrictions as either “threats” or “opportunities”, on problem orientation, coping behaviours, body image, and perceptions of eating behaviors and physical activity in a sample of female undergraduate students (*N* = 363). The group that reflected on challenges/barriers reported having a more negative problem orientation, being more negatively impacted by COVID-19, engaging in more maladaptive coping behaviors, and having less positive body image compared to participants who reflected on opportunities presented during the pandemic. Findings suggest that appraisals and problem orientation are malleable, and that people who tend to fixate on the challenges associated with COVID-19 may benefit from strategically reflecting on their own resilience and new opportunities that have arisen for engaging in health behaviors.

## 1. Introduction

The COVID-19 pandemic resulted in drastic changes to everyday life as mandatory lockdowns and social distancing measures were enforced to reduce spread of the virus. A growing body of literature has documented the adverse impact of the pandemic on physical and mental health, including elevated eating disorder (ED) symptoms and negative body image [1,2,3,4,5]. Rodgers [6] proposed that the disruptions to daily routines and the restrictions placed on activities (e.g., limited physical/outdoor activity, food insecurity, irregular eating habits, social isolation, limited access to treatment and other coping mechanisms) and the elevated dependency on media (e.g., videoconferencing) may be driving the increase in disordered eating.

Studies from large community samples suggest that many people perceive their eating behaviors, physical activity, and body image to have changed during the pandemic; however, large individual differences have been observed. For example, a British study reported that women, individuals under the age of 30, and those with a past/current eating disorder were disproportionately impacted by COVID-19 [7]. Women and those with an eating disorder were more likely to report greater difficulty regulating their eating, greater food preoccupation, more exercise, and worse body image during the pandemic. Younger individuals also reported worse body image and more thoughts about exercise. Another U.K. survey found that COVID-related anxiety was associated with increased body dissatisfaction and drive for thinness among women [5]. In an Italian survey, most people reported that their consumption of healthy foods had changed during lockdown; however, approximately an equal percentage of respondents reported that it had decreased (36%) versus increased (37%) [8]. Similarly, most Australian participants reported that their physical activity had changed, with 43% noting a decrease and 35% noting an increase [2].

One factor that may help account for the large individual variation in perceived changes to health behaviors and body image is a person’s cognitive appraisal of COVID-19 disruptions. According to Lazarus and Folkman’s influential transactional theory of stress and coping, an individual’s appraisal of a stressor has a significant impact on how they subsequently cope with stressful situations [9]. Challenge appraisals are associated with a perceived future gain and reflect an adaptive approach to stress whereby the individual views their circumstance as an opportunity for potential growth or positive change. In contrast, threat appraisals are associated with a perceived future harm or loss and are indicative of a maladaptive approach to stress whereby the individual emphasizes the situation’s potential negative implications.

Challenge and threat appraisals are also closely related to problem orientation. Problem orientation pertains to the cognitive schemas one holds to describe how they think or feel about a particular problem as well as their own ability to overcome the problem [10]. Positive problem orientation is an adaptive problem-solving approach where individuals tend to appraise problems as “challenges” or “opportunities”, anticipate positive outcomes, and feel confident in their ability to solve problems they encounter. Conversely, negative problem orientation is a maladaptive problem-solving approach where individuals are inclined to appraise stressful situations as “threats”, anticipate negative outcomes, and doubt their ability to overcome problems. As such, those who cognitively frame problems as positive “opportunities” are more likely to choose adaptive coping strategies than those who perceive problems as “threats” [10].

Within the context of COVID-19, people vary greatly in their subjective experience of the pandemic. Brose et al. examined the link between stress appraisals and negative experiences during the pandemic and found that threat appraisals were associated with greater negative affectivity and more frequent stressor occurrence [11]. Altogether, it is conceivable that one’s cognitive appraisal of challenges posed by the pandemic may also affect their coping strategies, health behaviors, and body image, which could help account for the large individual differences noted in previous studies.

The purpose of the present experimental study was to examine the impact of cognitive appraisals, specifically being prompted to reflect on the changes in health routines precipitated by COVID-19 restrictions as either “threats” or “opportunities”, on the following outcomes: (1) problem orientation, (2) perceived impact of and coping strategies used during COVID-19, and (3) body image. This is an important research question because cognitive appraisals are potentially modifiable; thus, the findings may inform the development of brief interventions to improve body image and coping. In consideration of the similarities between appraisal type (threat/opportunity) and problem orientation (negative/positive), the inclusion of a problem orientation measure was partly intended to serve as a manipulation check for the cognitive appraisal induction.

The following hypotheses were made:

**Hypothesis** **1.** *Problem Orientation: The Threat group will report a more negative problem orientation than the Opportunity group, and the Control group will fall intermediate between the two experimental groups.*

**Hypothesis** **2.** *COVID-19 Impact and Coping Strategies: The Threat group will report being more negatively impacted by COVID-19 and engaging in more maladaptive and less adaptive coping behaviors than the Opportunity group, and the Control group will fall intermediate between the two experimental groups.*

**Hypothesis** **3.** *Body Image: The Threat group will report having more negative body image and less positive body image than the Opportunity group, and the Control group will fall intermediate between the two experimental groups.*

## 2. Materials and Methods

### 2.1. Participants

Undergraduate women (*N* = 455) were recruited through the university psychology subject pool and were compensated with one credit (1%) towards their Introductory Psychology course. The inclusion criteria to participate included being female and a minimum of 17 years old. Men were excluded from the study because some of the body image measures were developed for use with women and have not been validated in male populations.

### 2.2. Measures

#### 2.2.1. Negative Problem Orientation Questionnaire (NPOQ)

The NPOQ [12] is a self-report measure of negative problem orientation. Respondents are presented with a list of 12 statements that evaluate the extent to which they tend towards a negative problem orientation when faced with everyday problems (e.g., “I see problems as a threat to my well-being”). Responses are rated on a Likert scale ranging from one (“not at all true of me”) to five (“extremely true”), with higher scores indicating a more negative problem orientation. The NPOQ has good internal consistency (α = 0.92) and high test-retest reliability (*r* = 0.80, *p* < 0.01) [12].

#### 2.2.2. Impact of COVID-19 Questionnaire

The Impact of COVID-19 Questionnaire is a self-report measure that was developed specifically for the present study to assess the extent to which COVID-19 has affected various aspects of health and health behaviors over the past three months. Respondents are asked to rate the perceived impact of COVID-19 on their eating habits, physical activity, self-care, and mental health on a scale ranging from one (“extremely negative impact”) to seven (“extremely positive impact”). Higher scores indicate a more positive impact. The scale had good internal consistency (α = 0.86) in the current sample and there is preliminary evidence for its convergent validity (i.e., significant negative correlations with negative problem orientation and maladaptive coping behaviors; significant positive correlations with general self-efficacy and adaptive coping behaviors).

#### 2.2.3. Coping during COVID-19 Questionnaire

The Coping During COVID-19 Questionnaire is a self-report measure that was developed specifically for the present study. It assesses an individual’s eating, physical activity, and self-care behaviors during COVID-19 over the past three months. Respondents are provided with a list of 15 statements and asked to rate how often they have experienced the thought or behavior identified in each statement over the past three months on a scale ranging from zero (“never”) to four (“all of the time”). Seven statements correspond with maladaptive coping (e.g., “Limited contact with others has led me to eat out of boredom”) and eight correspond with adaptive coping (e.g., “I’ve experienced a greater desire to take care of my body in order to remain healthy during the pandemic”). Higher scores on the maladaptive subscale indicate the presence of maladaptive coping strategies and higher scores on the adaptive subscale indicate the presence of adaptive coping strategies. Internal consistency was good for the maladaptive scale (α = 0.87) and adaptive scale (α = 0.80) in the current sample, and there is preliminary evidence for the scale’s convergent validity (i.e., adaptive coping is positively correlated with positive body image and negatively correlated with negative body image, whereas the reverse is true for maladaptive coping).

#### 2.2.4. Body Appreciation Scale-2 (BAS-2)

The BAS-2 [13] is a modified version of the Body Appreciation Scale (BAS) [14] that assesses positive body image. It includes 10 statements that assess respondents’ endorsement of favourable attitudes toward, and respect for, their bodies (e.g., “I feel good about my body”). Respondents indicate how often these statements are true about them on a scale ranging from one (“never”) to four (“always”). Higher scores indicate more positive body image. The BAS-2 has excellent internal consistency in women (α = 0.94), and good test-retest reliability [13].

#### 2.2.5. Body Image Acceptance and Action Questionnaire (BI-AAQ)

The BI-AAQ [15] is a self-report measure that evaluates body image flexibility. It is regarded as a measure of positive body image given its association with increased psychological flexibility, decreased body dissatisfaction, and less eating pathology. Respondents read 12 statements (e.g., “Worrying about my weight makes it difficult for me to live a life I value”) and asked to rate how often they feel this way on a scale from 1 (“never true”) to 7 (“always true”). Lower scores indicate greater body image flexibility. The BI-AAQ has shown high internal consistency (α = 0.92) and good test-retest reliability (*r* = 0.80, *p* < 0.01) [15].

#### 2.2.6. Body Shape Questionnaire-16A (BSQ-16A)

The BSQ-16A [16] is a shortened form of the Body Shape Questionnaire that assesses body image concerns. It includes 16 items (e.g., “Has eating even a small amount of food made you feel fat?”) and respondents report how often they have felt this way over the past month on a scale from one (“never”) to six (“always”). Total scores range from 16 to 96, with scores less than 38 representing an absence of body image concerns and scores at or above 66 representing marked body image concerns. The BSQ-16A has sound psychometric properties, including excellent internal consistency (α = 0.94) [17].

### 2.3. Procedure

This study was approved by the Institutional Research Ethics Board. Participants were recruited between September and December 2020 (which approximately coincided with the second wave of COVID-19) through an advertisement posted on the university psychology subject pool website. The study was conducted entirely online due to the COVID-19 pandemic. Following recruitment, participants were directed to Qualtrics (an online platform for data collection) and shown an informed consent form. Once consent was obtained, participants were randomized using Qualtrics to one of three experimental conditions in which they were prompted to engage in a 10-min writing exercise (approximately 500 words) about their eating behaviors, physical activity, and self-care during COVID-19, and the impact of these behaviors on their body image (see Appendix A for the instructions for each experimental condition): (1) the “Threat” condition prompted participants to reflect on any barriers posed by COVID-19 that may have hindered their ability to maintain good health and well-being; (2) the “Opportunity” condition prompted participants to reflect on any opportunities that COVID-19 presented for maintaining good health and well-being; and (3) the Control condition simply asked participants to reflect on their experience maintaining their health and well-being during COVID-19. Although each condition had different reflection prompts, all participants were asked to write about their eating behaviors, physical activity, and self-care during COVID, and their impact on body image.

Following the writing exercise, participants completed the following measures: (1) Impact of COVID-19 Questionnaire, (2) Coping During COVID-19 Questionnaire, (3) Negative Problem Orientation Questionnaire (NPOQ) [12], (4) Body Appreciation Scale-2 (BAS-2) [13], (5) Body Shape Questionnaire-16A (BSQ-16A) [16], (6) Body Image Acceptance and Action Questionnaire (BI-AAQ) [15], and (7) a demographic questionnaire. A debriefing form was provided upon completion of the questionnaires.

### 2.4. Statistical Analysis

The data were initially screened for missing data and to check if participants completed the writing task as instructed. Eighty-three participants were removed for making insufficient progress on the study that resulted in >10% of missing data (e.g., discontinuing the study prior to completion) and nine participants were removed for not following the specific instructions of the writing exercise (e.g., providing irrelevant or inappropriate responses). The statistical analyses were conducted with the remaining sample of *N* = 363. Nine of these participants had minimal missing data from the questionnaires. Mean imputation was used which has been found reliable in cases where 5% or less of the data is missing [18].

All statistical analyses were performed using SPSS Version 26 (International Business Machines Corporation [IBM], Armonk, NY, USA). Means (standard deviations) were computed for continuous variables and frequencies (%) were computed for categorical variables. Study hypotheses were tested using a series of one-way analyses of variance (ANOVA), with experimental condition (i.e., Threat; Opportunity; Control) as the independent variable, and the measures of negative problem orientation, impact of and coping during COVID-19, and body image as the dependent variables. Tukey post hoc tests were subsequently conducted to evaluate differences between groups.

## 3. Results

Participants (*N* = 363) had a mean age of 20.24 years (*SD* = 4.57), were ethnically diverse (but predominantly White/European; *N* = 107; 29.5%), predominantly identified as single/never married (*N* = 330; 90.9%), and many held part-time employment (*N* = 147; 40.5%). Participant characteristics are presented in Table 1.

### 3.1. Problem Orientation

Scores on each measure as a function of experimental condition are presented in Table 2. In support of Hypothesis 1, there was a significant difference between groups on the NPOQ, *F*(2, 360) = 3.52, *p* = 0.03, partial η^2^ = 0.019. A Tukey post hoc test indicated a significant difference between the Threat and Opportunity groups (*p* = 0.04). There was not a significant difference between the Threat and Control groups (*p* = 0.07) nor between the Opportunity and Control groups (*p* = 0.98). The Threat group (*M* = 35.91, *SE* = 1.07) scored significantly higher on the NPOQ than the Opportunity group (*M* = 32.41, *SE* = 1.04), meaning that they had a more negative problem orientation than the Opportunity group.

### 3.2. Impact of COVID-19

**Figure 1:**
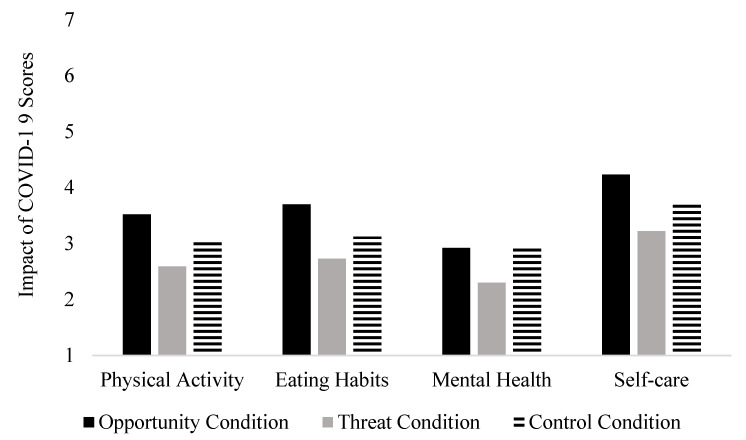
Perceived Impact of COVID-19 on Health Behaviors as a Function of Experimental Condition. *Note.* 1 = extremely negative impact; 4 = neutral impact; 7 = extremely positive impact.

In support of Hypothesis 2, there was a significant difference between groups on the Impact of COVID-19 Questionnaire, *F*(2, 360) = 10.88, *p* < 0.001, partial η^2^ = 0.057. A Tukey post-hoc test indicated a significant difference between the Opportunity and Threat groups (*p* < 0.001), as well as between the Threat and Control groups (*p* = 0.02). There was not a significant difference between the Opportunity and Control groups (*p* = 0.11). The Opportunity group scored significantly higher on the Impact of COVID-19 Questionnaire than those in the Threat group, meaning that their health behaviors were not as negatively impacted by COVID-19. Those in the Threat group scored significantly lower on the Impact of COVID-19 Questionnaire than those in the Control group, meaning that their health behaviors were more negatively impacted by COVID-19 than the other two groups. Mean scores for the Physical Activity, Eating Habits, Mental Health, and Self-Care items of the Impact of COVID-19 questionnaire are presented in Figure 1.

### 3.3. Coping during COVID-19

In partial support of Hypothesis 2, there was a significant difference between groups on the Maladaptive Coping subscale of the Coping During COVID-19 Questionnaire, *F*(2, 359) = 6.554, *p* < 0.001, partial η^2^ = 0.05. A Tukey post hoc test indicated a significant difference between the Threat and Opportunity groups (*p* = 0.001), as well as between the Threat and Control groups (*p* < 0.001). There was not a significant difference between the Opportunity and Control groups (*p* = 0.98). The Threat group (*M* = 22.99, *SE* = 0.57) scored significantly higher than the Opportunity group (*M* = 19.87, *SE* = 0.57) and the Control group (*M* = 19.72, *SE* = 0.62) on the Maladaptive Coping subscale of the Coping During COVID-19 Questionnaire, meaning that they reported more maladaptive coping mechanisms than the other two groups. However, there was not a significant difference between groups on the Adaptive Coping subscale of the Coping During COVID-19 Questionnaire, *F*(2, 359) = 10.01, *p* = 0.07, partial η^2^ = 0.014.

### 3.4. Body Image

Overall, there was partial support for Hypothesis 3 across various measures of body image and related constructs.

#### 3.4.1. Body Appreciation

There was a significant difference between groups on the BAS-2, *F*(2, 360) = 3.29, *p* = 0.04, partial η^2^ = 0.018. A Tukey post hoc test indicated a marginal significant difference between the Threat and Opportunity groups *(p* = 0.059). There were no significant differences between the Threat and Control groups (*p* = 0.08) or between the Opportunity and Control groups (*p* = 0.99). The Threat group (*M* = 30.25, *SE* = 0.86) scored somewhat lower on the BAS-2 than the Opportunity group (*M* = 33.07, *SE* = 0.79), meaning that they had less body appreciation than the Opportunity group.

#### 3.4.2. Body Image Flexibility

There was a significant difference between groups on the BI-AAQ, *F*(2, 360) = 6.056, *p* = 0.003, partial η^2^ = 0.033. A Tukey post hoc test indicated a significant difference between the Threat and Opportunity groups *(p* = 0.03), as well as between the Threat and Control groups (*p* = 0.003). There was not a significant difference between the Opportunity and Control groups (*p* = 0.71). The Threat group (*M* = 47.09, *SE* = 1.69) scored significantly higher on the BI-AAQ than the Opportunity group (*M* = 41.12, *SE* = 1.67) and Control group (*M* = 39.24, *SE* = 1.65), meaning that they had less body image flexibility than the other two groups.

#### 3.4.3. Body Dissatisfaction

There was a significant difference between groups on the BSQ-16A, *F*(2, 360) = 5.06, *p* = 0.007, partial η^2^ = 0.027. A Tukey post hoc test indicated a significant difference between the Threat and Control groups (*p* = 0.006). There were no significant differences between the Threat and Opportunity groups (*p* = 0.10) or between the Opportunity and Control groups (*p* = 0.56). The Threat group (*M* = 53.14, *SE* = 1.74) scored significantly higher on the BSQ than the Control group (*M* = 45.31, *SE* = 1.79), meaning that they had more negative body image than the Control group.

## 4. Discussion

An emerging body of research indicates that COVID-19 has had a significant impact on eating behaviors, physical activity, and body image; however, substantial individual differences in the trajectories of these changes suggest that the relationship is complex [7]. The aim of the present study was to examine the impact of participants’ cognitive appraisal of COVID-19 on self-reported problem orientation, perceived impact of COVID-19 on health behaviors, coping behaviors, and body image. Participants who were prompted to reflect on the difficulties or barriers they faced reported having a more negative problem orientation, being more negatively impacted by COVID-19, engaging in more maladaptive coping behaviors, and having less body image flexibility than those who made opportunity appraisals. Conversely, participants who made opportunity appraisals reported having a more positive problem orientation, being less negatively impacted by COVID-19, engaging in less maladaptive coping behaviors, and having greater body appreciation and body image flexibility in comparison to those who made threat appraisals. An important implication of this research is that cognitive appraisals are modifiable; therefore, individuals with a negative problem orientation who are inclined to appraise stressors as threats may benefit from brief training to adopt a more positive problem orientation by emphasizing opportunities that have arisen.

In support of the hypotheses, the group that was prompted to reflect on opportunities presented by COVID-19 had a less negative problem orientation than participants prompted to reflect on barriers encountered during COVID-19. This finding is consistent with literature on problem orientation which suggests that those with a more positive problem orientation are more likely to cognitively appraise stressors as “opportunities”, whereas those with a more negative problem orientation have a tendency to perceive challenges as “threats” [10]. While problem orientation is considered consistent over time in that it reflects one’s general tendency to appraise problems as either opportunities or threats, it is also malleable. For instance, individuals with anxiety and depressive disorders typically have a negative problem orientation, and one of the functions of cognitive behavioral therapy (CBT) is to encourage individuals to adopt a more positive problem orientation [19]. Interestingly, women tend to have a more negative problem orientation than men [12], which may help account for the increased prevalence of anxiety and depressive disorders among women. The present finding serves as a manipulation check in that being instructed to make threat appraisals did in fact prompt those participants to adopt a more negative problem orientation. Unexpectedly, those who made opportunity appraisals did not differ from the Control group in self-reported ratings of negative problem orientation. One plausible explanation for this finding is that the NPOQ was created to specifically measure negative problem orientation. As such, its validity and reliability as a measure of positive problem orientation is limited and it cannot be assumed that scoring low on negative problem orientation indicates a positive problem orientation [12].

In support of the hypotheses, the group that reflected on barriers reported being more negatively impacted by COVID-19 and engaging in more maladaptive coping behaviors than the group that was prompted to reflect on opportunities. Given that participants were randomly assigned to groups and thus presumably did not differ with respect to eating behaviors, physical activity, self-care, and mental health at baseline, this finding suggests that the way an individual appraises COVID-19 restrictions can influence one’s perception of the impact of COVID-19 and their engagement in health behaviors.

The null finding for adaptive coping could potentially be due to the undetermined validity and reliability of the Adaptive Coping subscale given that this measure was developed specifically for the current study. However, another plausible explanation is that individuals had reduced access to healthy coping behaviors due to COVID-19 restrictions [6]. For example, store and gym closures may have limited opportunities for engaging in healthy behaviors such as eating a nutritious diet and exercising. Furthermore, social distancing measures may have exacerbated this impact by deterring individuals from seeking social support.

There was partial support for the hypothesis that cognitive appraisals would impact body image. Those who made opportunity appraisals reported greater body image appreciation and body image flexibility than those who made threats appraisals. However, the two groups did not differ with respect to body dissatisfaction, which may suggest that being prompted to reflect on opportunities during COVID-19 had a stronger impact on variables associated with positive body image. Positive and negative body image are conceptualized as separate constructs, meaning they should not be regarded as opposite ends of the same spectrum [20] and the current findings support this distinction.

While the lack of group difference in body dissatisfaction was inconsistent with our hypotheses, perhaps those who reflected on opportunities presented by COVID-19 were engaging in healthy behaviors (e.g., physical activity), but to an unhealthy extent (e.g., overly strict exercise regimens) due to weight and shape concerns. Previous research indicates that women and individuals with a current/past eating disorder were more likely to report increasing their physical activity during COVID-19 [7]. Another explanation for this null finding may come from research by Keel et al. which found that students in general judged themselves as gaining more weight during the pandemic despite minimal changes in actual weight [21]. According to cut-offs on the Body Shape Questionnaire-16A [16], the Opportunity and Control groups reported “mild” shape concerns, and the Threat group reported “moderate” shape concerns.

### Limitations and Future Directions

Some strengths of this study include the ethnically diverse participant sample, the experimental study design, and the use of validated measures of problem orientation and body image and related constructs. However, several limitations must be considered when interpreting the findings. First, the Impact of COVID-19 and Coping During COVID-19 measures were specifically developed for the current study given the absence of existing validated COVID-19 measures when the study was conceptualized. Second, participants were exclusively female undergraduate students, and the majority had a healthy BMI, so it will be important to replicate this research in a larger and more representative community sample. It would also be informative to replicate this study in a sample of individuals with disordered eating and body image concerns to examine whether cognitive reappraisal is a helpful strategy for focusing on one’s strengths, bolstering self-efficacy, and improving body image given the well documented adverse impact of COVID-19 in this group [1,2,7]. Third, the outcome measures were administered immediately following the 10-min writing exercise, so the durability of the effects over a longer follow-up period are unknown.

If the findings are replicated, the information gained could be used to inform the development of a brief intervention to shift people’s perceptions regarding the impact of COVID-19, their own ability to cope with the changes, and their body image. Although speculative at this point given that the current study did not assess participants’ problem orientation prior to the writing task, such an intervention could be particularly beneficial for individuals who have a negative problem orientation. If a single 10-min reflective writing exercise has an immediate impact, then perhaps people could be encouraged to keep a journal over a longer period to reflect on what they did to maintain healthy habits and any obstacles they overcame. Exercises focused on practicing gratitude (e.g., keeping a gratitude journal; identifying “three good things” that happened and what caused them) have been shown to improve emotional, psychological, and social well-being [22].

## 5. Conclusions

COVID-19 restrictions have had a significant impact on health behaviors and body image; however, large individual differences exist. Although COVID-19 has presented many challenges for engaging in health behaviors, it has also afforded some new opportunities. The findings of the current study suggest that focusing on difficulties or barriers encountered may result in greater perceived negative impact of COVID-19, more maladaptive coping behaviors, and less positive body image compared to focusing on opportunities. Fortunately, cognitive appraisals and problem orientation are modifiable, and encouraging people to reflect on their own resilience and opportunities that have arisen during COVID-19 may result in more adaptive coping behaviors and more positive body image. Beyond the realm of body image, findings also provide insight into how adaptive cognitive appraisals regarding COVID-19—that is, viewing the pandemic as an opportunity for positive change and growth as opposed to perceiving it solely as a threat to one’s wellbeing (i.e., posttraumatic growth) [23]—may in turn facilitate healthier coping mechanisms during the pandemic.

## Figures and Tables

**Table 1:** Participant Characteristics as a Function of Experimental Condition.

Variable	Positive-Opportunity (*n* = 120)	Negative-Threat (*n* = 122)	Control (*n* = 121)	Full Sample (*N* = 363)
BMI (kg/m^2^)	22.84 (4.54)	23.65 (4.69)	22.96 (4.43)	22.96 (4.43)
Age (y)	20.04 (4.27)	20.37 (4.73)	20.28 (4.70)	20.24 (4.57)
Ethnicity	*N* (%)	*N* (%)	*N* (%)	*N* (%)
Arab/West Asian	8 (6.7)	8 (6.6)	13 (10.7)	29 (8.0)
Black/African/Caribbean	6 (5.0)	6 (4.9)	5 (4.1)	17 (4.7)
White/European	33 (27.5)	39 (32.0)	35 (28.9)	107 (29.5)
East Asian	14 (11.7)	12 (9.8)	9 (7.4)	35 (9.6)
Indigenous	-	-	1 (0.8)	1 (0.3)
Latin/South American	4 (3.3)	4 (3.3)	5 (4.1)	13 (3.6)
South Asian	20 (16.7)	22 (18.0)	26 (21.5)	68 (18.7)
Southeast Asian	12 (10.0)	18 (14.8)	12 (9.9)	42 (11.6)
Other	18 (15.0)	12 (9.8)	15 (12.4)	45 (12.4)
Prefer not to specify	4 (3.3)	1 (0.8)	-	5 (1.4)
Marital Status	*N* (%)	*N* (%)	*N* (%)	*N* (%)
Married	2 (1.7)	3 (2.5)	3 (2.5)	8 (2.2)
Common-law	2 (1.7)	6 (4.9)	4 (3.3)	12 (3.3)
Single/never married	113 (94.2)	106 (86.9)	111 (91.7)	330 (90.9)
Prefer not to specify	3 (2.5)	7 (5.7)	3 (2.5)	13 (3.6)
Employment Status	*N* (%)	*N* (%)	*N* (%)	*N* (%)
Full-time	10 (8.3)	14 (11.5)	15 (12.4)	39 (10.7)
Part-time	49 (40.8)	46 (37.7)	52 (43.0)	147 (40.5)
Unemployed	31 (25.8)	38 (31.1)	33 (27.3)	102 (28.1)
Social assistance	25 (20.8)	21 (17.2)	19 (15.7)	65 (17.9)
Disability support	1 (0.8)	-	-	1 (0.3)
Prefer not to specify	4 (3.3)	3 (2.5)	2 (1.7)	9 (2.5)

Note. Results are means (standard deviations) unless otherwise specified. There were no significant differences in participant characteristics across groups.

**Table 2:** Mean Scores and Standard Deviations on Dependent Variables as a Function of Experimental Condition.

Variable	Positive-Opportunity		Negative-Threat		Control	
	*M*	*SD*	*M*	*SD*	*M*	*SD*
Problem Orientation	32.41 ^a^	11.40	35.91 ^b^	11.77	32.69 ^a,b^	11.13
Impact of COVID	14.38 ^b^	5.54	10.84 ^a^	6.04	12.84^b^	6.12
Coping During COVID—Adaptive Coping	23.33	5.40	21.70	5.50	22.20	6.11
Coping During COVID—Maladaptive Coping	19.87 ^a^	6.17	22.99 ^b^	6.27	19.72 ^a^	6.83
Body Appreciation	33.07 ^b^	8.69	30.25 ^a^	9.49	32.88 ^a,b^	10.48
Body Image Inflexibility	41.12 ^a^	18.26	47.09 ^b^	18.70	39.24 ^a^	18.13
Body Dissatisfaction	47.91 ^a,b^	19.83	53.14 ^b^	19.22	45.31 ^a^	19.64

Note. Superscripts ^a,b^ are used to denote significant differences between groups according to Tukey post hoc tests (*p* < 0.05). Means with a superscript ^a^ are significantly lower than means with a superscript ^b^. Means with the same superscript are not significantly different from one another.

## Data Availability

The datasets that support the findings of this study are available from the corresponding author upon request.

## Appendix A. Writing Exercise Instructions

### Appendix A.1. Opportunity Condition

The global pandemic of COVID-19 resulted in sudden and drastic changes in many aspects of life. Due to physical distancing requirements, direct social contact was limited to those in one’s household and only essential services were allowed to remain open, which meant that schools, recreational facilities, and most businesses and workplaces were closed.

Although COVID-19 posed many challenges, living through a global pandemic may also increase one’s sense of personal resiliency. These challenges may have presented opportunities for practicing adaptive coping strategies, and developing innovative ways of maintaining good physical health, well-being, and self-esteem despite the restrictions posed by COVID.

We would like you to reflect on your personal experiences during COVID-19. We are interested in learning about any healthy routines you were able to start or continue in order to maintain good physical health, well-being, and self-esteem during COVID, including any strategies you used to overcome challenges posed by COVID restrictions.

Using the space provided below, take the next 10 min or so to write about any opportunities you’ve had for establishing and/or maintaining healthy routines during COVID-19 with respect to eating behaviors, physical activity, and self-care. Please also describe the ways in which these opportunities have impacted your body image.

### Appendix A.2. Threat Condition

The global pandemic of COVID-19 resulted in sudden and drastic changes in many aspects of life. Due to physical distancing requirements, direct social contact was limited to those in one’s household and only essential services were allowed to remain open, which meant that schools, recreational facilities, and most businesses and workplaces were closed.

COVID-19 posed many challenges. These challenges may have made it more difficult to practice adaptive coping strategies, and posed barriers to maintaining good physical health, well-being, and self-esteem due to the restrictions posed by COVID.

We would like you to reflect on your personal experiences during COVID-19. We are interested in learning about any difficulties you experienced in maintaining good physical health, well-being, and self-esteem during COVID, including any barriers or challenges you faced due to the restrictions posed by COVID.

Using the space provided below, take the next 10 min or so to write about any difficulties or challenges you’ve experienced for establishing and/or maintaining healthy routines during COVID-19 with respect to eating behaviors, physical activity, and self-care. Please also describe the ways in which these challenges have impacted your body image.

### Appendix A.3. Control Condition

The global pandemic of COVID-19 resulted in sudden and drastic changes in many aspects of life. Due to physical distancing requirements, direct social contact was limited to those in one’s household and only essential services were allowed to remain open, which meant that schools, recreational facilities, and most businesses and workplaces were closed.

We would like you to reflect on your personal experiences during COVID-19. Using the space provided below, take the next 10 min or so to write about your physical care routines during COVID-19 with respect to eating behaviors, physical activity, and self-care. Please also describe the ways in which these routines impacted your body image.
